# Immunosuppressant Drug Specific Risk of Malignancy After Organ Transplantation: A Population-Based Analysis of Texas Medicare Beneficiaries

**DOI:** 10.3390/cancers17132161

**Published:** 2025-06-26

**Authors:** Luca Cicalese, Jordan R. Westra, Zachary C. Walton, Yong-Fang Kuo

**Affiliations:** 1 Division of Transplant Surgery, Department of Surgery, University of Texas Medical Branch, 6.120 John Sealy Annex, 301 University Boulevard, Galveston, TX 77555, USA; 2 Department of Biostatistics & Data Science, University of Texas Medical Branch, Galveston, TX 77555, USA; jrwestra@utmb.edu (J.R.W.); yokuo@utmb.edu (Y.-F.K.); 3 John Sealy School of Medicine, University of Texas Medical Branch, Galveston, TX 77555, USA; zcwalton@utmb.edu; 4 Sealy Center on Aging, University of Texas Medical Branch, Galveston, TX 77554, USA

**Keywords:** immunosuppressant, mycophenolate mofetil, cyclosporine, sirolimus, tacrolimus, organ transplant, malignancy

## Abstract

**Background/Objectives:** Prolonged use of immunosuppressive drugs (IMDs) is correlated with increased risk of cancer in transplant patients. However, detailed side-by-side analysis of cancer risk associated with individual IMDs in the same population is not available. The aim of this study was to identify drug-specific risks of cancer for commonly used transplant IMDs. **Methods:** We analyzed the risk of cancer for the IMDs commonly used in transplant patients (tacrolimus (TAC), cyclosporin (CY), sirolimus (SIR), mycophenolate (MMF), and their combinations) in Texas Medicare beneficiaries between 2007 and 2018. **Results:** Of 7721 transplant recipients receiving an IMD of interest, 2261 developed cancer. There was an increased risk of any cancer diagnosis with the use of TAC (HR: 1.49; 95% CI: 1.25–1.78) and CY (HR: 1.51; 95% CI: 1.19–1.92), and decreased risk with use of MMF (HR: 0.77; 95% CI: 0.67–0.89). Cancer-specific models revealed increased risk of liver cancer (HR: 5.25, 95% CI: 2.03–13.61) and decreased risk of ovarian/uterine cancer (HR: 0.25, 95% CI: 0.07–0.84) with TAC; increased risk of lung cancer with CY (HR: 5.06, 95% CI: 1.47–17.41); and increased risk of lymphoma associated with SIR (HR: 2.80, 95% CI: 1.00–7.81). **Conclusions:** TAC increases cancer risk, and MMF decreases cancer risk. Individual cancer types also vary in risk associated with individual IMDs. This study provides new information on IMD-specific cancer risk that can guide individualized screening/treatment decisions to reduce the risk associated with specific cancers after transplantation.

## Simple Summary

Though it has commonly been accepted that chronic IMD use increases the risk of cancer, the subject is largely understudied as we do not know cancer risk safety profiles for individual IMDs or individual cancer types. We analyzed the risk of cancer for the IMDs commonly used in transplant patients (tacrolimus, cyclosporin, sirolimus, mycophenolate, and their combinations) in Texas Medicare beneficiaries and from the Texas Cancer Registry between 2007 and 2018. The results of this study are vital for understanding long-term adverse effects of cancer with IMD use and highlight the importance of individualizing treatment regimens to minimize cancer risk using medications with lower risk of long-term harm. For example, TAC is one of the most commonly used IMDs in transplant but may confer the highest risk of cancer compared to other transplant IMDs according to this study.

## 1. Introduction

Recent advances in immunosuppressive drugs (IMDs) including the development of new or more potent drugs have expanded the clinical indications for IMD usage [1,2,3,4,5]. Additionally, the individual effects of IMDs on the development of cancer has not been analyzed in detail. Most patients chronically receiving IMDs are organ transplant recipients. These patients are kept on lifelong immunosuppression therapy with various IMDs to prevent rejection, enhance graft survival, and improve quality of life [6,7,8]. Substantial benefits in terms of quality of life and mortality have been shown with the use of these treatments in organ transplant recipients. However, immunosuppression results in reduced immuno-surveillance for cancer and an increased risk of infections. Certain viral infections observed with a higher frequency in immunosuppressed patients have been associated with higher risk of malignancy [9,10,11]. Specific cancers associated with these infections include lymphomas (Epstein–Barr virus), cervical cancer (human papillomavirus), Kaposi’s sarcoma (human herpes virus-8, human immunodeficiency virus), and liver cancer (human hepatitis virus B and C) [12,13,14,15,16,17].

Cancers not associated with viral infections are also present in immunosuppressed patients. For example, non-melanoma skin cancer is reportedly the most common de novo malignancy following liver and kidney transplants [18]. Prolonged use of IMDs in transplant patients have previously been associated with an increased risk of malignancies [18,19]. However, these studies analyze cumulative immunosuppression and fail to compare individual drugs or the relationship with length of exposure to specific IMDs or IMD combinations. For instance, we recently determined that even short-term exposure to IMDs increases the risk of cancer [20].

The increasing prevalence of IMDs in treating non-transplant related conditions, such as rheumatoid arthritis, lupus, myasthenia gravis, interstitial lung disease, fibromyalgia, inflammatory bowel syndrome, and other autoimmune diseases, necessitates a more profound, granular analysis of the cancer risk associated with IMD use. To date, a few studies observing the risks of malignancy attributed to IMD use have focused on a small number of individual IMD drugs. For example, increased risk of non-melanoma skin cancer, lymphoma, and lymphoproliferative disorders have been linked to use of azathioprine or 6-mercaptopurine [21,22,23]. Furthermore, a study examining risk of malignancy from methotrexate in the treatment of rheumatoid arthritis revealed a higher risk of all malignancies except liver cancer [24].

Although all IMDs ultimately result in immunosuppression, individual drugs can affect cancer development in different ways. Currently, the relative risks attributable to the use of different IMDs, or the mechanism associated with such risks, are poorly understood.

Furthermore, cancer has different incidence rates geographically. How these variations are affected by individual IMDs also warrants a better understanding of this phenomenon. For example, Texas ranks among the leading US states for incidence of liver cancer (hepatocellular carcinoma) with an incidence rate 26–37% higher than the national rate [25]. This finding is believed to be associated with Texas’ predominant role in the petrochemical and agricultural industries as Texas is home to the second largest petrochemical industry and agricultural industry in the nation. Furthermore, Texas residents living near these industries, and especially residents involved in occupations related to these industries, are exposed to higher levels of potentially dangerous pollutants. Recently, we identified a higher incidence of liver cancer in Texas counties associated with higher air levels of various chemical pollutants [26]. We also observed a consistent, significant, positive association between the incidence of liver cancer and hepatitis C prevalence rates with increased exposure to vinyl chloride [27]. Additionally, prior studies have demonstrated that, in Texas, proximity to an oil refinery is associated with increased risk of various types of cancer [28].

Considering these factors, a state like Texas, that presents a peculiar incidence of certain types of cancer when compared to other states or countries, warrants the need for a specific, detailed analysis of the effects individual IMDs have on cancer progression. The organ transplant recipients in Texas, being among the most common and chronic users of IMD, will benefit from such an analysis that should eventually be extended to other states to analyze their peculiarity nationally, for which information is currently not available. Therefore, we analyzed the effect of the most common IMD used in transplantation (tacrolimus (TAC), cyclosporine (CY), sirolimus (SIR), and mycophenolate (MMF)) on the development of cancer in Texas transplant patients.

## 2. Materials and Methods

### 2.1. Data Selection

This study was approved by the UTMB Institutional Review Board (IRB—22-031) and utilized Medicare claims data from a dataset of 100% of Texas Medicare beneficiaries. In this study, the following Medicare files were used: Master Beneficiary Summary File (MBSF), Medicare Provider Analysis and Review (MedPAR) files, Outpatient Standard Analytic (OutSAF) files, Medicare Carrier files, and Part D Event (PDE) files. MBSF files were used for information on beneficiary characteristics; MedPAR, outpatient, and carrier files were used to extract diagnosis and procedure information; PDE files were used to extract prescription drug information. Information from these databases are depersonalized, and individual patients cannot be identified. Furthermore, neither the patients nor the public had any involvement in the study design or any subsequential processes relating to this study.

Beneficiaries who had organ transplants between 2008 and 2018 were included in the initial cohort. Transplants were defined by procedure codes from MedPAR, (utSAF, and Carrier claims files) [20]. From this cohort, beneficiaries were excluded if they did not have continuous Medicare fee-for-service coverage (hospital (part A), medical (part B), and prescription (part D) or coverage with Health Maintenance Organization enrollment) for the 12 months prior to organ transplant, or if they had a cancer diagnosis in the 12 months before transplant.

### 2.2. Data Curation

The primary outcome was a new diagnosis of cancer and was determined by International Classification of Disease, Ninth or Tenth Revision (ICD-9 or ICD-10) codes found in any MedPAR, OutSAF, or Carrier claims files [20]. The primary independent variables of interest were the type of IMD taken, classified as tacrolimus, sirolimus, cyclosporine, mycophenolate, or other (including IMDs not typically associated with organ transplant). Receipt of an IMD was ascertained by filled prescriptions using national drug codes (NDC) from the Part D Event (PDE) or for injections using healthcare common procedure coding system (HCPCS) codes (Supplemental Table S1). To account for the potential for changing IMD, each drug type was measured daily following organ transplant as a time-dependent variable. Because hospital-administered medications are not available in the Medicare PDE database, assessment of IMD use began following discharge from the hospital.

Other variables of interest for assessment of association with cancer diagnosis included sex, age at transplant (<40, 40–49, 50–59, 60–69, 70–79, 80+ years), race (White, Black, Hispanic, Other), Medicare original entitlement reason (old age or disabled/end-stage renal disease (ESRD), Medicaid dual eligibility (yes/no), Elixhauser comorbidity index count (0–2, 3–5, 6–7, 8–9, 10+), ESRD diagnosis (yes/no), secondary IMD indication (yes/no), viral hepatitis (yes/no), and receipt of other medications (yes/no individually—anti-bacterial, anti-viral, anti-fungal, electrolytes, metformin). These fixed variables were all measured at baseline with demographic information obtained from the Medicare Beneficiary Summary file.

### 2.3. Data Analysis

Descriptive characteristics were calculated for the overall transplant cohort and by IMD drug type. While the analytic models included IMD type as a time-dependent variable, the data in Table 1 are presented by the first IMD type used for each person following hospital discharge. To assess the association of IMD types with the development of cancer, a Fine and Gray proportional hazards model was used, with time to cancer diagnosis as the dependent variable and death as a competing event. Beneficiaries were censored if they lost Medicare coverage or at the end of the study period (31 December 2019). Each of the IMD types were included as individual variables on a time-dependent basis, allowing for drug type to change and multiple drug types to be used on the same day. Assessing IMD type daily allows for those who switch drugs or use multiple drugs to contribute to the risk attributed to each IMD type. Including the drugs as individual variables isolates the risk associated with each IMD type by adjusting for use of other drugs daily. Four models were created under these conditions—model 1: IMD types only; model 2: model 1 + demographics; model 3: model 2 + Elixhauser comorbidity and IMD indication; model 4: model 3 + other medications. Additionally, model 4 was used to assess the interaction between IMD types and age and race, as well as the interaction between TAC and MMF. For significant interactions, model 4 (minus the strata variable) was used to calculate stratum-specific hazards ratios for IMD types. Cancer-specific hazard ratios were also calculated for liver, kidney, skin, ovarian/uterine, prostate, colorectal, lung, and breast cancers, as well as lymphoma. Each model for a specific cancer was created independent of other cancer types, and other cancer types were not considered as censoring or competing events. All analyses were performed using SAS (v9.4, Cary, NC, USA).

## 3. Results

The cohort consisted of 7721 Medicare beneficiaries who received an organ transplant between 2008 and 2018. In the cohort, the mean age was 51.3 (standard deviation (SD): 15.7), 45.2% were women, 89.3% were eligible for Medicare due to old age, and 52.0% were eligible for Medicaid. The most common comorbid conditions were renal failure (76.5%), hypertension (75.3%), anemia (68.0%), and fluid/electrolyte disorders (66.0%) (Table 1). The follow-up period extended through 31 December 2019. The percentage of the cohort eligible was 96.2% at 6 months, 91.1% at 1 year, and 65.2% at 3 years. Most patients in our study receiving IMD for transplantation were White (46.9%), while the rest were Black (18.8%), Hispanic (17.5%), and Other (16.8%). This distribution is similar to the ethnic distribution described in the 2020 Census for the population in Texas (White 61.6%, Black 12.4%, Hispanic 18.7%, Other 7.3%).

Based on first used IMD, TAC (77.2%), and MMF (76.5%) were the most used drugs, followed by CY (5.2%), SIR (2.3%), and other (5.0%). A combination of any of the drugs was used 72.2% of the time, with the most common combination being TAC and MMF (67.6%). The percentages of use were similar over time (Table 2).

An association between specific IMD types and cancer was observed. In Fine and Gray proportional hazards regression (Table 3) of the association between IMD type and cancer, CY (hazard ratio (HR): 1.51, 95% confidence interval (CI): 1.19, 1.92) and TAC (HR: 1.49, 95% CI: 1.25, 1.78) were shown be associated with an increased risk of cancer diagnosis, while MMF (HR: 0.77, 95% CI: 0.67, 0.89) was associated with a decreased risk of cancer. Use of SIR or other IMD types were not associated with cancer diagnosis (Table 3). The interaction between TAC and MMF was significant (*p* = 0.0246) and showed increased risk for cancer diagnosis (OR: 1.21, 95% CI: 1.03–1.44).

The age analysis showed, as expected, an increased risk of cancer associated with advanced age (HR 2.13 at 60–69; HR 3.30 at 70–79 and HR 2.31 > 80 years old compared to <40 years old). The models testing for interaction between IMD types and age showed that the interactions with age were not significant.

The models testing for interaction between IMD types and race showed that the interactions with race were significant. Stratified analysis showed that increased risk of cancer for CY or TAC use persisted only among those who were White (Table 4). Among Black and Hispanic patients, there were no significant differences for any of the drugs.

For cancer-specific results (Supplemental Table S2), the shortest time to cancer was among those who developed liver cancer (0.71 years, 95% CI: 0.53, 0.89) and the longest was among those who developed breast cancer (1.98 years, 95% CI: 1.67, 2.30). In the cancer-specific models (Table 5), TAC was associated with increased risk of liver cancer (HR: 5.25, 95% CI: 2.03, 13.61) and decreased risk of ovarian/uterine cancer (HR: 0.25, 95% CI: 0.07, 0.84); CY was associated with increased risk of lung cancer (HR: 5.06, 95% CI: 1.47, 17.41), and SIR was associated with increased risk of lymphoma (HR: 2.80, 95% CI: 1.00, 7.81).

## 4. Discussion

The use of IMDs in organ transplantation to prevent organ rejection is well established. It has been previously established that chronic IMD treatment after organ transplantation increases the risk of developing cancer compared to the general population [29,30,31,32,33,34]. This increase is believed to be primarily associated with reduced immunosurveillance against tumors and viral infections, typically seen with a higher frequency in these patients, that are associated with the development of certain cancers.

We analyzed, for the first time, the entire Medicare patient population of the State of Texas by identifying all patients who received an organ transplantation and were treated for at least one year with the most used IMDs (TAC, CY, MMF, and SIR). Individual analysis of individual IMDs, or a combination of IMDs, revealed that the risk of certain cancers differs significantly depending on the medication used.

Different IMDs commonly used in transplant patients have a common denominator: the chronic suppression of the immune system (i.e., T and B cell activity). However, different IMDs accomplish this effect via different mechanisms and have varying potency, tissue accumulation, and distribution. All these factors potentially affect the risk, type, and localization of malignancy. Thus, to better understand our findings, one must consider the differences between the mechanism of action (MOA), potency, and organ/tissue distribution of each IMD.

Calcineurin inhibitors (CNIs), such as TAC and CY, exert their immunosuppressive effects by reducing interleukin-2 (IL-2) production and IL-2 receptor expression, which reduces overall T-cell activation. TAC inhibits T-lymphocyte activation [35]. The MOA of CY is similar, except that the binding protein is different (cyclophilin rather than calmodulin). TAC is 10–100 times more potent than CY in its immunosuppressive effects and thus is more potent and effective than CY [36,37]. TAC’s potency possibly explains its increased HR. Additionally, CY’s high HR observed can be explained by the fact that clinically, when CY is used, the dose is adjusted to reach a similar immunosuppressive effect to TAC, and therefore its relative weakness is, at least in part, compensated. Furthermore, evidence from animal models suggests that CY itself may promote cancer progression via production of transforming growth factor beta (TGF- β). In vitro, treatment of a normally noninvasive adenocarcinoma cell line with CY induced an invasive phenotype and in immunodeficient animals, while CY promoted tumor growth [38].

If IMDs can directly promote cancer, one must consider their tissue distribution. Highest tissue concentrations of CY are observed in the thymus, spleen, lymph nodes, bone marrow, liver, pancreas, kidneys, adrenal glands, lungs, and skin. TAC accumulates mainly in the lungs, spleen, heart, kidneys, brain, muscles, and pancreas [39,40,41,42,43,44,45,46].

Interestingly, our study identified an increased risk of cancer associated with CNI use in patients who self-identified their racial background as White compared to those who self-identified as Black (Table 4). These observed racial differences could be explained by the known racial and ethnic differences in the pharmacokinetics and pharmacodynamics of CNIs. For example, it has been shown that African Americans have a higher frequency of CYP3A5 expression, which leads to lower TAC blood concentrations. Contrarily, White individuals of European origin have a higher frequency of CYP3A4 variants that slow CNI metabolism [47,48].

CY’s possible direct carcinogenic effects may contribute to the HRs of cancers observed in certain organs in our analysis. The high rate of lung cancer (HR 5.06) observed with CY, for example, could be a result of CY’s possible direct carcinogenic effects combined with its preferential accumulation in this organ. Although not statistically significant in our analysis, CY was also associated with an increased HR of liver (HR 2.7) and skin (HR 2.61) cancer, both sites of accumulation of the drug. TAC also showed a high rate of liver cancer (HR 5.25) and a rapid time to cancer (0.71 years from use) but does not accumulate significantly within this organ. It is important to consider that in this analysis, a previous diagnosis of cancer was a criterion of exclusion, and therefore these are only de novo cancers and not recurrences such as those observed after liver transplant performed for liver cancer (hepatocellular carcinoma and cholangiocarcinoma). This could be due to the MOA differences between TAC and CY. In fact, among the organs where TAC accumulates, the HR cancer progression was low, and there is no evidence in animal models, unlike CY, that TAC directly causes cancer. TAC is also excreted in the biliary tree, which may explain its association with liver cancer, but this has not been investigated.

Another consideration is that Texas has a high rate of liver cancer in the general population, even without the use of IMD or transplant, as previously discussed [25,26,27,28]. Although this specific subset of transplanted, immunosuppressed patients was not analyzed by geographical distribution or by association with the level of pollutant distribution, the use of TAC (and not the other IMDs) appears to further exacerbate the risk of liver cancer.

It is more difficult to explain the lower rate of ovarian/uterine cancer (HR 0.25) observed in transplant patients treated with TAC. Interestingly, TAC is an effective inhibitor of P-glycoprotein [49]; it has a potentially profound role in reversing multidrug resistance and is used in conjunction with anticancer therapy in ovarian cancer [50]. Currently, there is no evidence of TAC exerting a direct anticancer effect. However, these are estrogen-dependent cancers [51], which hormone has the opposite effect of TAC by increasing IL-2 levels and can raise TAC blood levels. Therefore, additional mechanistic studies on IL-2 and other possible correlations are needed to better elucidate the protection exerted by TAC on ovarian cancer seen in our study.

MMF, another commonly used IMD in organ transplantation, binds avidly to serum albumin [52,53]. Its MOA does not suppress IL-2 like CNIs do but instead involves mycophenolic acid. MMF blocks the replication of activated T-lymphocytes during S-phase, thereby favoring induction of apoptosis, and suppresses primary antibody responses more efficiently than secondary responses [49].

MMF also has synergistic activity with viral inhibitors of DNA polymerase and reverse transcription. This antiviral activity could offer protection against viral-associated cancers such as lymphoma (EBV) and liver cancer (HCV). For example, renal transplant recipients treated with CY and MMF had a significantly lower rate of EBV infections than those receiving CY alone [54].

MMF’s potential cancer protective activity may also stem from its effects on both T-cell and tumor cell adhesion to endothelial cells. This suggests that MMF not only interferes with the invasion of allo-activated T-cells (protective in organ rejection) but might also be of value in managing post-transplantation malignancy [55]. Moreover, several studies suggest that MMF could offer an anti-cancer effect and be even used as an anticancer agent [56,57,58,59,60,61,62]. Therefore, the relatively lower risk of cancer observed with MMF in our analysis can be explained by the anti-cancer effect of this drug, as demonstrated by previous studies. Moreover, its antiviral effect can further contribute to lowering the risk of cancer by reducing viral infections, such as EBV, that have been frequently associated with the development of post-transplant lymphoma or HCV-related liver cancer. These specific neoplasms had a low HR when patients were receiving MMF (Lymphoma HR 0.65; and liver cancer HR 0.68). This is particularly interesting for liver cancer when considering the high risk of developing this tumor in Texas. Additionally, MMF’s protective effect was more prominent in Hispanic patients (Table 4). This may be explained by the fact that these patients potentially experience higher MMF exposure compared to other ethnic groups. This observation has been linked to variations in uridine diphosphate glucuronosyltransferase (UGT) enzyme genotypes, which play a critical role in MPA metabolism [63,64].

SIR has immunosuppressive, antifungal, and anticancer properties by interrupting several cellular signal transduction pathways via the inhibition of the mammalian TOR (mTOR) receptor. SIR inhibits T-lymphocyte proliferation and inhibits IL-2-dependent and -independent proliferation of B lymphocytes [65]. SIR also affects the proliferation of non-lymphoid tumor cells [66,67]. In vitro, in vivo, and clinical trial experiments have shown that inhibition of mTOR has therapeutic efficacy against cancer [68,69,70,71,72,73]. The tissue of distribution for SIR is primarily the intestines, liver, spleen, heart, lung, and kidney [74,75]. Our analysis observed a high HR for lymphoma in patients using SIR (HR: 2.80, 95% CI: 1.00, 7.81), which can be explained by SIR’s anti-cancer properties being limited to non-lymphoma cancer. In fact, we observed a low HR for most tumors other than lymphoma, liver, and skin cancer when patients were treated with SIR (Table 5).

The slightly elevated HRs observed for liver and skin cancer with SIR were not statistically significant. There is evidence that SIR can improve survival after liver transplant for advanced liver cancer; however, this study did not show a decrease in recurrence rate of liver cancer [76]. Therefore, the potential curative or preventive effect of SIR on liver cancer remains unclear. Previous studies show a possible protective effect of SIR on skin cancer after transplant [77,78]. However, these studies involve patients with a previous history of skin cancer that developed while receiving TAC who switched to SIR after diagnosis. In this study, all patients with a preexisting diagnosis of cancer prior to the beginning of IMD use were excluded. Though SIR might reduce cancer development via inhibition of angiogenesis, its prevention of de novo skin cancer formation in a state like Texas with high ultraviolet exposure throughout the year also remains unclear.

This study has some limitations. Firstly, we cannot ascertain whether or not the patients actually take the prescriptions, as we analyzed the PDE reflecting what was prescribed and filled. Second, the indications of prescription might not be completely captured from the linkage between PDE and medical claims files. Third, socioeconomic factors, environment, and health behaviors could be important confounders of the association between type of IMD and cancer incidence. Additionally, demographic information is presumed to have been reported directly by patients to their respective healthcare entities that report information to the Texas Medicare database. Fourth, the severity of comorbid conditions, which could modify the risk of cancer, is not available from Medicare data. Fifth, we did not examine the influence of IMD dosage on the risk of cancer, which may also influence cancer risk. Finally, our findings may not be generalized to patients with Medicare advantage care or to patients living outside of Texas.

This study analyzes in detail the cancer risk of individual IMDs used for organ transplantation in Texas. Furthermore, this study displays, for the first time, a side-by-side comparison of cancer risk using different IMDs in the same transplant patient population (controlled for comorbidities and demographics) and demonstrates unique risks and benefits of different IMDs, some of which differ from the current data. This study provides important information to support treatment preferences, to reduce the risk of cancer after transplant, and provide a foundation for a larger national analysis.

## 5. Conclusions

In conclusion, our study of Texas transplant recipients comparing the commonly used IMD in transplant recipients indicates an increased risk of cancer varied by type of IMDs. Specifically, CNIs (TAC and CY) are associated with higher risk. The risk was lower with MMF or its association. SIR use had low hazard in the early period, but the hazard grew at a faster rate over time than the other drug types. We also observed a higher risk in White people, while the risk was not significantly affected by age. Different cancer patterns were observed with different IMDs. TAC was associated with increased risk of liver cancer and decreased risk of ovarian/uterine cancer; CY was associated with increased risk of lung cancer; and SIR was associated with increased risk of lymphoma.

Therefore, this study suggests that different IMDs have a different impact of cancer risk post-transplant. Clinically, this information could be used to develop a personalized screening protocol and immunosuppressive regimen for transplant patients based on the risk factors of various cancers. Therefore, a more careful selection of IMDs; screening for preexisting liver conditions that can increase the risk of liver cancer especially when using TAC and CY; and the development of a more focused and aggressive neoplastic diagnostic screening are strongly encouraged.

## Figures and Tables

**Table 1:** Beneficiary characteristics, overall and by first immunosuppression drug type used.

	All		Tacrolimus		Sirolimus		Cyclosporine		Mycophenolate		Other	
All	7721	100%	5963	77%	181	2%	399	5%	5909	77%	388	5%
**Transplant year**												
2008	646	8.4%	423	7.1%	73	40.3%	83	20.8%	453	7.7%	51	13.1%
2009	652	8.4%	456	7.6%	46	25.4%	58	14.5%	497	8.4%	36	9.3%
2010	627	8.1%	461	7.7%	30	16.6%	58	14.5%	467	7.9%	42	10.8%
2011	635	8.2%	485	8.1%	13	7.2%	46	11.5%	457	7.7%	27	7.0%
2012	649	8.4%	513	8.6%	6	3.3%	31	7.8%	485	8.2%	27	7.0%
2013	661	8.6%	476	8.0%	4	2.2%	49	12.3%	486	8.2%	48	12.4%
2014	734	9.5%	580	9.7%	1	0.6%	27	6.8%	561	9.5%	37	9.5%
2015	734	9.5%	586	9.8%	1	0.6%	15	3.8%	560	9.5%	34	8.8%
2016	742	9.6%	656	11.0%	1	0.6%	12	3.0%	639	10.8%	29	7.5%
2017	736	9.5%	633	10.6%	3	1.7%	8	2.0%	617	10.4%	29	7.5%
2018	905	11.7%	694	11.6%	3	1.7%	12	3.0%	687	11.6%	28	7.2%
**Sex**												
Male	4228	54.8%	3378	56.6%	110	60.8%	244	61.2%	3341	56.5%	216	55.7%
Female	3493	45.2%	2585	43.4%	71	39.2%	155	38.8%	2568	43.5%	172	44.3%
**Age**												
Mean, SD	51.3	15.7	50.0	14.8	49.4	13.3	51.3	15.1	49.6	14.9	50.2	20.9
<40	1809	23.4%	1502	25.2%	37	20.4%	86	21.6%	1507	25.5%	97	25.0%
40–49	1461	18.9%	1208	20.3%	49	27.1%	87	21.8%	1235	20.9%	49	12.6%
50–59	1783	23.1%	1461	24.5%	47	26.0%	89	22.3%	1445	24.5%	75	19.3%
60–69	1827	23.7%	1361	22.8%	41	22.7%	94	23.6%	1310	22.2%	113	29.1%
70–79	759	9.8%	419	7.0%	7	3.9%	43	10.8%	400	6.8%	49	12.6%
80+	82	1.1%	12	0.2%	0	0.0%	0	0.0%	12	0.2%	5	1.3%
**Beneficiary Race Code**												
White	3620	46.9%	2652	44.5%	76	42.0%	178	44.6%	2599	44.0%	232	59.8%
Black	1451	18.8%	1176	19.7%	31	17.1%	93	23.3%	1160	19.6%	65	16.8%
Other	1297	16.8%	1010	16.9%	10	5.5%	45	11.3%	1010	17.1%	55	14.2%
Hispanic	1353	17.5%	1125	18.9%	64	35.4%	83	20.8%	1140	19.3%	36	9.3%
**Original Reason for Entitlement Code**												
Old Age	6897	89.3%	5538	92.9%	171	94.5%	347	87.0%	5537	93.7%	316	81.4%
Disability/ESRD	824	10.7%	425	7.1%	10	5.5%	52	13.0%	372	6.3%	72	18.6%
**Dual Eligibility**												
No	3709	48.0%	2766	46.4%	82	45.3%	195	48.9%	2697	45.6%	189	48.7%
Yes	4012	52.0%	3197	53.6%	99	54.7%	204	51.1%	3212	54.4%	199	51.3%
**Elixhauser Count**												
Mean, SD	7.4	4.0	7.5	3.9	8.8	3.0	8.5	3.4	7.6	3.9	7.2	3.8
0–2	1037	13.4%	723	12.1%	5	2.8%	14	3.5%	710	12.0%	40	10.3%
3–5	1059	13.7%	756	12.7%	16	8.8%	42	10.5%	714	12.1%	79	20.4%
6–7	1582	20.5%	1277	21.4%	38	21.0%	96	24.1%	1258	21.3%	92	23.7%
8–9	1779	23.0%	1442	24.2%	51	28.2%	116	29.1%	1461	24.7%	67	17.3%
10+	2264	29.3%	1765	29.6%	71	39.2%	131	32.8%	1766	29.9%	110	28.4%
**Comorbidities**												
AIDS/HIV	69	0.9%	58	1.0%	3	1.7%	1	0.3%	56	0.9%	1	0.3%
Alcohol Abuse	235	3.0%	170	2.9%	10	5.5%	22	5.5%	167	2.8%	11	2.8%
Blood Loss Anemia	660	8.5%	526	8.8%	15	8.3%	40	10.0%	534	9.0%	24	6.2%
Cardiac Arrhythmia	2537	32.9%	1889	31.7%	65	35.9%	180	45.1%	1851	31.3%	163	42.0%
Chronic Pulmonary Disease	1805	23.4%	1298	21.8%	44	24.3%	127	31.8%	1234	20.9%	174	44.8%
Coagulopathy	1597	20.7%	1239	20.8%	34	18.8%	104	26.1%	1168	19.8%	85	21.9%
Congestive Heart Failure	2107	27.3%	1609	27.0%	49	27.1%	161	40.4%	1617	27.4%	103	26.5%
Deficiency Anemia	5253	68.0%	4379	73.4%	153	84.5%	283	70.9%	4397	74.4%	207	53.4%
Depression	1326	17.2%	936	15.7%	25	13.8%	79	19.8%	889	15.0%	97	25.0%
Diabetes Complicated	3338	43.2%	2611	43.8%	98	54.1%	166	41.6%	2605	44.1%	139	35.8%
Diabetes Uncomplicated	3051	39.5%	2507	42.0%	101	55.8%	131	32.8%	2509	42.5%	104	26.8%
Drug Abuse	156	2.0%	107	1.8%	7	3.9%	11	2.8%	107	1.8%	26	6.7%
Fluid and Electrolyte Disorders	5092	66.0%	4059	68.1%	145	80.1%	312	78.2%	4063	68.8%	251	64.7%
Hypertension complicated	5816	75.3%	4518	75.8%	161	89.0%	328	82.2%	4493	76.0%	270	69.6%
Hypertension Uncomplicated	5712	74.0%	4703	78.9%	167	92.3%	328	82.2%	4769	80.7%	204	52.6%
Hypothyroidism	1559	20.2%	1145	19.2%	43	23.8%	102	25.6%	1167	19.7%	89	22.9%
Liver Disease	1731	22.4%	1368	22.9%	63	34.8%	113	28.3%	1343	22.7%	100	25.8%
Obesity	1508	19.5%	1193	20.0%	16	8.8%	59	14.8%	1172	19.8%	63	16.2%
Other Neurological Disorders	1020	13.2%	702	11.8%	33	18.2%	81	20.3%	731	12.4%	67	17.3%
Paralysis	155	2.0%	80	1.3%	5	2.8%	8	2.0%	79	1.3%	12	3.1%
Peptic Ulcer Disease excluding bleeding	229	3.0%	175	2.9%	10	5.5%	17	4.3%	178	3.0%	18	4.6%
Peripheral Vascular Disorders	2173	28.1%	1681	28.2%	54	29.8%	111	27.8%	1672	28.3%	86	22.2%
Psychoses	261	3.4%	172	2.9%	13	7.2%	16	4.0%	163	2.8%	14	3.6%
Pulmonary Circulation Disorders	802	10.4%	583	9.8%	9	5.0%	72	18.0%	562	9.5%	92	23.7%
Renal Failure	5905	76.5%	4889	82.0%	174	96.1%	338	84.7%	4947	83.7%	228	58.8%
Rheumatoid Arthritis/collagen	794	10.3%	566	9.5%	16	8.8%	48	12.0%	576	9.7%	56	14.4%
Valvular Disease	1851	24.0%	1369	23.0%	46	25.4%	143	35.8%	1345	22.8%	107	27.6%
Viral Hepatitis	900	11.7%	740	12.4%	16	8.8%	60	15.0%	744	12.6%	35	9.0%
Weight Loss	1051	13.6%	765	12.8%	41	22.7%	75	18.8%	775	13.1%	68	17.5%
**Any Indication for IMD Use**	6815	88.3%	5551	93.1%	176	97.2%	326	81.7%	5578	94.4%	289	74.5%
ESRD	6574	85.1%	5464	91.6%	174	96.1%	314	78.7%	5505	93.2%	236	60.8%
Secondary Indication	1032	13.4%	734	12.3%	21	11.6%	61	15.3%	738	12.5%	79	20.4%
**Cancer Diagnosis**												
Diagnosis to match TCR	2261	29.3%	1704	28.6%	53	29.3%	145	36.3%	1623	27.5%	139	35.8%
Malignant Neoplasm	1885	24.4%	1366	22.9%	43	23.8%	126	31.6%	1282	21.7%	124	32.0%
In Situ, Uncertain, or Unspecified Behavior	1176	15.2%	946	15.9%	24	13.3%	48	12.0%	932	15.8%	65	16.8%
**Died in Follow-up**	290	3.8%	108	1.8%	10	5.5%	16	4.0%	89	1.5%	8	2.1%
**Other Drugs Used**												
Antibacterial	6938	89.9%	5520	92.6%	164	90.6%	362	90.7%	5447	92.2%	353	91.0%
Antiviral	6411	83.0%	5430	91.1%	104	57.5%	306	76.7%	5391	91.2%	316	81.4%
Antifungal	3809	49.3%	2984	50.0%	133	73.5%	200	50.1%	2974	50.3%	280	72.2%
Electrolyte	458	5.9%	327	5.5%	15	8.3%	28	7.0%	298	5.0%	29	7.5%
Metformin	899	11.6%	683	11.5%	16	8.8%	31	7.8%	677	11.5%	43	11.1%

**Table 2:** Immunosuppression drug type use by time.

IMD Type	Baseline		1 Year		3 Years		5 Years	
Tacrolimus	5963	77.2%	5441	77.3%	3555	70.6%	1539	58.6%
Sirolimus	181	2.3%	296	4.2%	234	4.6%	135	5.1%
Cyclosporine	399	5.2%	406	5.8%	266	5.3%	145	5.5%
Mycophenolate	5909	76.5%	5120	72.8%	3205	63.6%	1348	51.3%
Other	388	5.0%	629	8.9%	399	7.9%	168	6.4%
Any combination	5575	72.2%	5121	72.8%	3236	64.2%	1379	52.5%
Tacrolimus and mycophenolate	5218	67.6%	4743	67.4%	2925	58.1%	1213	46.2%
Total	7721	100%	7035	100%	5037	100%	2628	100%

**Table 3:** Cancer diagnosis hazard ratios, unadjusted and adjusted for demographics, comorbidities, and medication usage.

		Unadjusted HR (95% CI)	Demographics Adjusted HR (95% CI)	Comorbidity Adjusted HR (95% CI)	Medication Adjusted HR (95% CI)
**IMD Type**	Cyclosporine	1.19 (0.95, 1.48)	1.40 (1.11, 1.77)	1.50 (1.19, 1.91)	1.51 (1.19, 1.92)
	Mycophenolate *	0.66 (0.58, 0.75)	0.73 (0.63, 0.84)	0.76 (0.65, 0.87)	0.77 (0.67, 0.89)
	Other	0.93 (0.76, 1.13)	0.90 (0.74, 1.10)	0.90 (0.73, 1.11)	0.91 (0.74, 1.12)
	Sirolimus	0.68 (0.49, 0.95)	0.90 (0.64, 1.27)	0.95 (0.67, 1.35)	1.05 (0.74, 1.49)
	Tacrolimus *	1.13 (0.98, 1.30)	1.39 (1.18, 1.63)	1.47 (1.23, 1.76)	1.49 (1.25, 1.78)
**Sex**	Female		Ref	Ref	Ref
	Male		0.97 (0.88, 1.06)	1.02 (0.92, 1.13)	1.06 (0.95, 1.18)
**Age**	<40		Ref	Ref	Ref
	40–49		1.06 (0.89, 1.27)	1.01 (0.85, 1.21)	1.01 (0.84, 1.21)
	50–59		1.30 (1.10, 1.53)	1.22 (1.03, 1.44)	1.23 (1.04, 1.46)
	60–69		2.24 (1.93, 2.61)	2.10 (1.80, 2.45)	2.13 (1.82, 2.49)
	70–79		3.44 (2.82, 4.20)	3.26 (2.67, 3.98)	3.30 (2.70, 4.04)
	80+		2.36 (1.54, 3.60)	2.24 (1.47, 3.44)	2.31 (1.51, 3.52)
**Race**	White		Ref	Ref	Ref
	Black		0.81 (0.70, 0.93)	0.82 (0.71, 0.94)	0.87 (0.76, 1.01)
	Other		1.41 (1.24, 1.61)	1.13 (0.96, 1.34)	1.11 (0.94, 1.31)
	Hispanic		0.83 (0.71, 0.96)	0.84 (0.72, 0.98)	0.88 (0.75, 1.03)
**Original Entitlement**	Old Age		Ref	Ref	Ref
	Disability/ESRD		0.81 (0.68, 0.96)	0.79 (0.66, 0.94)	0.78 (0.65, 0.94)
**Medicaid Eligible**	No		Ref	Ref	Ref
	Yes		0.80 (0.72, 0.88)	0.79 (0.72, 0.88)	0.79 (0.71, 0.87)
**Elixhauser Comorbidity Count**	0–2			Ref	Ref
	3–5			0.63 (0.51, 0.78)	0.64 (0.51, 0.79)
	6–7			0.63 (0.52, 0.77)	0.62 (0.50, 0.76)
	8–9			0.67 (0.55, 0.82)	0.66 (0.54, 0.81)
	10+			0.68 (0.57, 0.82)	0.64 (0.53, 0.78)
**ESRD**				0.86 (0.73, 1.02)	0.87 (0.73, 1.03)
**Secondary IMD Indication**				0.99 (0.85, 1.14)	0.93 (0.80, 1.07)
**Viral Hepatitis**				1.28 (1.11, 1.48)	1.30 (1.13, 1.51)
**Medications**	Anti-bacterial				1.32 (1.20, 1.45)
	Anti-viral				1.79 (1.56, 2.06)
	Anti-fungal				1.17 (1.00, 1.37)
	Electrolytes				1.01 (0.73, 1.38)
	Metformin				1.51 (1.18, 1.94)

* Interaction between tacrolimus and mycophenolate was significant (*p* = 0.0246), OR: 1.21, 95% CI: 1.03–1.44. Reference criteria for corresponding HRs among demographics are designated by Ref.

**Table 4:** Adjusted hazard ratios by race.

IMD Type	White	Black	Hispanic	Other
Cyclosporine	2.10 (1.52, 2.92)	1.00 (0.54, 1.86)	0.91 (0.48, 1.73)	1.27 (0.62, 2.63)
Mycophenolate	0.90 (0.72, 1.13)	1.35 (0.92, 1.99)	0.40 (0.30, 0.52)	0.77 (0.52, 1.12)
Other IMD	1.11 (0.85, 1.44)	0.97 (0.52, 1.82)	0.25 (0.10, 0.60)	0.15 (0.02, 1.09)
Sirolimus	1.59 (1.00, 2.52)	1.25 (0.50, 3.14)	0.42 (0.08, 2.27)	0.67 (0.31, 1.44)
Tacrolimus	2.28 (1.76, 2.96)	1.00 (0.65, 1.53)	0.84 (0.63, 1.14)	1.25 (0.69, 2.28)

**Table 5:** Cancer-specific hazard ratios by cancer type and immunosuppression drug type.

Cancer Type	Parameter	Medication Adjusted HR (95% CI)
Breast	Cyclosporine	0.83 (0.25, 2.77)
	Mycophenolate	1.59 (0.70, 3.63)
	Other	1.26 (0.38, 4.22)
	Sirolimus	1.08 (0.25, 4.63)
	Tacrolimus	0.60 (0.27, 1.31)
Colorectal	Cyclosporine	1.89 (0.53, 6.82)
	Mycophenolate	1.49 (0.47, 4.66)
	Other	1.10 (0.23, 5.21)
	Sirolimus	0.00 (0.00, 0.00)
	Tacrolimus	0.79 (0.26, 2.42)
Kidney	Cyclosporine	0.30 (0.07, 1.31)
	Mycophenolate	1.53 (0.81, 2.89)
	Other	0.25 (0.03, 1.85)
	Sirolimus	0.87 (0.20, 3.84)
	Tacrolimus	0.66 (0.35, 1.22)
Liver	Cyclosporine	2.67 (0.66, 10.86)
	Mycophenolate	0.68 (0.38, 1.22)
	Other	0.13 (0.02, 1.00)
	Sirolimus	1.94 (0.39, 9.63)
	Tacrolimus	5.25 (2.03, 13.61)
Lung	Cyclosporine	5.06 (1.47, 17.41)
	Mycophenolate	0.99 (0.38, 2.63)
	Other	2.46 (0.90, 6.71)
	Sirolimus	0.00 (0.00, 0.00)
	Tacrolimus	1.49 (0.51, 4.35)
Lymphoma	Cyclosporine	0.27 (0.06, 1.16)
	Mycophenolate	0.65 (0.38, 1.11)
	Other	0.95 (0.36, 2.53)
	Sirolimus	2.80 (1.00, 7.81)
	Tacrolimus	0.60 (0.34, 1.05)
Ovarian/Uterine	Cyclosporine	0.63 (0.06, 6.25)
	Mycophenolate	0.78 (0.16, 3.82)
	Other	0.00 (0.00, 0.00)
	Sirolimus	0.00 (0.00, 0.00)
	Tacrolimus	0.25 (0.07, 0.84)
Prostate	Cyclosporine	1.57 (0.34, 7.40)
	Mycophenolate	0.92 (0.37, 2.25)
	Other	0.30 (0.04, 2.16)
	Sirolimus	0.00 (0.00, 0.00)
	Tacrolimus	1.86 (0.55, 6.24)
Skin	Cyclosporine	2.61 (0.99, 6.87)
	Mycophenolate	1.26 (0.58, 2.73)
	Other	0.83 (0.28, 2.47)
	Sirolimus	2.94 (0.90, 9.61)
	Tacrolimus	1.70 (0.78, 3.71)

## Data Availability

The data underlying this article was provided by the Centers for Medicare and Medicaid Services and the Texas Cancer Registry under Data Use Agreements. Data requests should be made to these entities.

## Supplementary Materials

The following supporting information can be downloaded at: https://www.mdpi.com/article/10.3390/cancers17132161/s1, Table S1: Codes utilized for collection and identification of data; Table S2: Average time to cancer in years by cancer type.

## Abbreviations

CNIs (calcineurin inhibitors); CY (cyclosporine); ESRD (end-stage renal disease); HCPCS (healthcare common procedure coding system); IL-2 (interleukin-2); IMD (immunosuppressive drug); MBSF (Master Beneficiary Summary File); MedPAR (Medicare Provider Analysis and Review); MMF (mycophenolate mofetil); MOA (mechanism of action); mTOR (mammalian TOR); NDC (National Drug Codes); NF-AT (nuclear factor of activated T-cells); OutSAF (Outpatient Standard Analytic); PDE (Part D Event); SD (standard deviation); SIR (sirolimus); TAC (tacrolimus); TGF-**β** (transforming growth factor beta).
