# Supplementary material for: Immunosuppressant Drug Specific Risk of Malignancy After Organ Transplantation: A Population-Based Analysis of Texas Medicare Beneficiaries
Source: Cancers (Basel). 2025 Jun 26;17(13):2161. doi: 10.3390/cancers17132161 (PMC12249258; doi:10.3390/cancers17132161)
Supplement: Supplementary file 1 [file cancers-17-02161-s001.zip › cancers-3618602-supplementary.pdf]

Supplemental Table S1. Codes utilized for collection and identification of data.

Desired Information	Codes Used
Transplants	ICD-9-V3: 00.91, 00.92, 00.93, 07.94, 11.6, 11.60, 11.69, 33.50, 33.5, 33.51, 33.52, 33.6, 37.51, 41.00, 41.0, 41.01, 41.02, 41.03, 41.04, 41.05, 41.06, 41.07, 41.08, 41.09, 41.91, 41.94, 46.97, 49.74, 50.5, 50.51, 50.59, 52.80, 52.8, 52.82, 52.83, 52.84, 52.85, 52.86, 55.6, 55.69, 63.53, 65.92, 82.56, 82.58, 83.75, 83.77, 86.64; ICD-10-PCS: first position 0 and third position Y; HCPCS: 32850, 32851, 32852, 32853, 32854, 32855, 32856, 33927, 33928, 33929, 33930, 33933, 33935, 33940, 33944, 33945, 38240, 38241, 38242, 44135, 44136, 47133, 47135, 47136, 47140, 47141, 47142, 47143, 47144, 47145, 47146, 47147, 48160, 48550, 48551, 48552, 48554, 48556, 50300, 50320, 50323, 50325, 50327, 50328, 50329, 50340, 50360, 50365, 50370, 50380
New Diagnosis of Cancer	ICD-9: 140.x-172.x, 173.0, 173.10, 173.19, 173.20, 173.29, 173.30, 173.39, 173.40, 173.49, 173.50, 173.59, 173.60, 173.69, 173.70, 173.79, 173.80, 173.89, 173.9, 173.90, 173.99, 174.x-208.x, 209.0, 209.1, 209.2, 209.30, 209.31, 209.32, 209.33, 209.34, 209.35, 209.36, 209.7, 225.x, 227.3, 227.4, 228.1, 228.2, 230.x-234.x, 237.0, 237.1, 237.5, 237.6, 237.9, 238.4, 238.7, 239.6, 239.7, 273.3, 277.89; ICD-10: C00.x-C43.x, C44.0, C44.10, C44.19, C44.20, C44.29, C44.30, C44.39, C44.40, C44.49, C44.50, C44.59, C44.60, C44.69, C44.70, C44.79, C44.80, C44.89, C44.9, C44.90, C44.99, C45.x-C96.x, D00.x-D09.x, D18.2, D32.x, D33.x, D35.2, D35.3, D35.4, D42.x, D43.x, D44.3, D44.4, D44.5, D45, D46.x, D47.1, D47.2, D47.3, D47.4, D47.9, D49.6, D49.7, R85.614, R87.614, R87.624

Supplemental Table S2. Average time to cancer in years by cancer type.

Cancer Type	N	Years to Cancer (95% CI)
Breast	56	1.98 (1.67, 2.30)
Colorectal	44	1.75 (1.43, 2.07)
Kidney	98	1.25 (1.03, 1.46)
Liver	103	0.71 (0.53, 0.89)
Lung	40	1.75 (1.38, 2.11)
Lymphoma	83	1.10 (0.86, 1.33)
Ovarian/Uterine	13	1.06 (0.55, 1.56)
Prostate	61	1.59 (1.33, 1.86)
Skin	78	1.96 (1.71, 2.21)
Total	2,037	2.04 (1.96, 2.12)
